# Supplementary material for: Biostimulating Gut Microbiome with Bilberry Anthocyanin Combo to Enhance Anti-PD-L1 Efficiency against Murine Colon Cancer
Source: Microorganisms. 2020 Jan 25;8(2):175. doi: 10.3390/microorganisms8020175 (PMC7074734; doi:10.3390/microorganisms8020175)
Supplement: Supplementary file 1 [file microorganisms-08-00175-s001.pdf]

Supporting Information

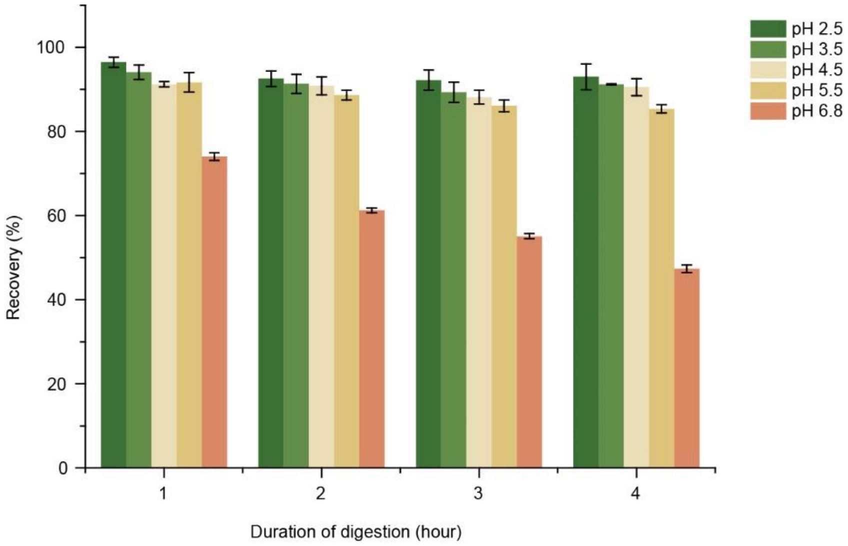

Figure S1. Total anthocyanin content during incubation at different pHs.

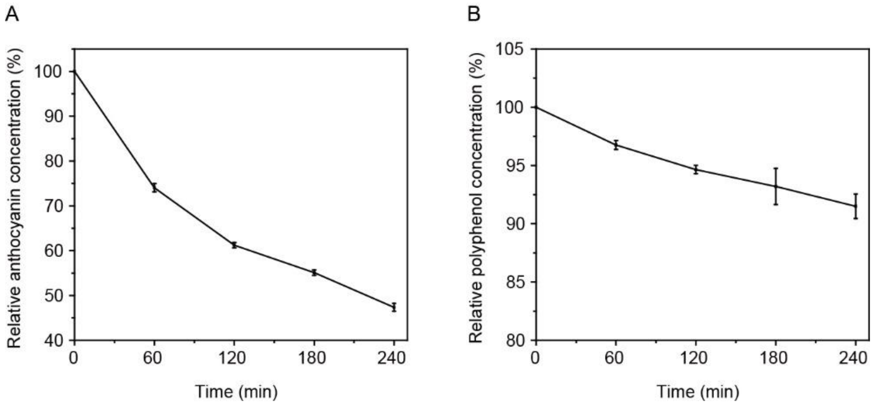

Figure S2. Stability of bilberry anthocyanins in simulated intestinal fluid without pancreatin at pH 6.8 for 4 h. Total anthocyanin (A) and polyphenol (B) content during incubation.

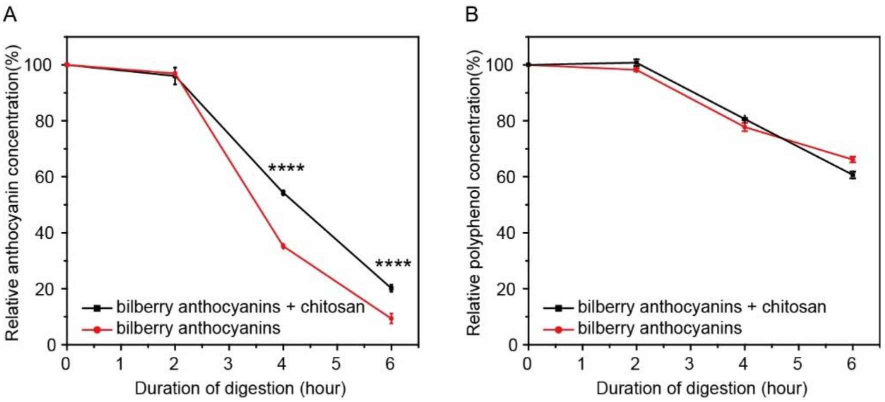

Figure. S3. Relative content of anthocyanins (A) and polyphenols (B) of bilberry anthocyanins and anthocyanin-chitosan in simulated gastric fluid with pepsin at pH 2.0 for 2 h and subsequently in simulated intestinal fluid (SIF) with pancreatin at pH 7.5 for 4 h. The concentrations of chitosan and bilberry extracts were 1 mg/mL and 0.5 mg/mL, respectively.

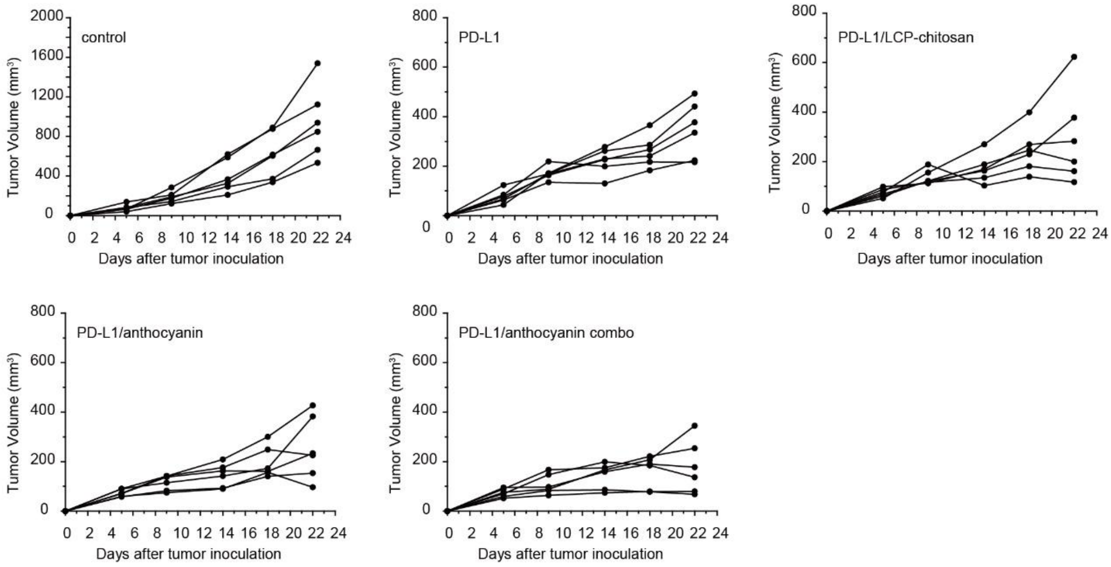

Figure S4. Individual tumor size growth data for MC38 tumor-bearing mice in each group.

Table S1. Total polyphenol and anthocyanin content before and after incubation in simulated gastric fluid without pepsin (pH 1.2).

Time (min)	Total anthocyanin recovery (%)	Total polyphenol recovery (%)
0	100	100
60	104.46±0.36	99.94±0.81
120	103.08±1.03	100.89±1.40

Table S2. Adonis variance analysis based on Bray–Curtis distance matrices at OTU level.

Group	R^2	P-value
CT vs. PD	0.27571	0.033
PD vs. BA	0.28997	0.027
PD vs. PE	0.29733	0.020
PD vs. BP	0.30182	0.030
PE vs. BP	0.40326	0.031
